# Obstructive sleep apnea combined dyslipidemia render additive effect on increasing atherosclerotic cardiovascular diseases prevalence

**DOI:** 10.1186/s12944-016-0267-7

**Published:** 2016-05-26

**Authors:** Zhiyong Cao, Ping Zhang, Zhiqing He, Jing Yang, Chun Liang, Yusheng Ren, Zonggui Wu

**Affiliations:** Shanghai Changzheng Hospital, Second Military Medical University, Shanghai, 200003 China; Branch of No. 411 Hospital, PLA, Shanghai, 200003 China; Shanghai Changhai Hospital, Second Military Medical University, Shanghai, 200003 China

**Keywords:** Dyslipidemia, Obstructive sleep apnea, Atherosclerotic cardiovascular diseases

## Abstract

**Background:**

Current study was designed to investigate the effects of obstructive sleep apnea (OSA) combined dyslipidemia on the prevalence of atherosclerotic cardiovascular diseases (ASCVD).

**Methods:**

This was a cross-sectional study and subjects with documented dyslipidemia and without previous diagnosis of OSA were enrolled. Polysomnography was applied to evaluate apnea-hypopnea index (AHI). Based on AHI value, subjects were classified into four groups: without OSA, mild, moderate and severe OSA groups. Clinical characteristics and laboratory examination data were recorded. Relationship between AHI event and lipid profiles was analyzed, and logistic regression analysis was used to evaluate the effects of OSA combined dyslipidemia on ASCVD prevalence.

**Results:**

Totally 248 subjects with dyslipidemia were enrolled. Compared to the other 3 groups, subjects with severe OSA were older, male predominant and had higher smoking rate. In addition, subjects with severe OSA had higher body mass index, waist-hip ratio, blood pressure, and higher rates of overweight and obesity. Serum levels of fasting plasma glucose, glycated hemoglobin, LDL-C and CRP were all significantly higher. ASCVD prevalence was considerably higher in subjects with severe OSA. AHI event in the severe OSA group was up to 35.4 ± 5.1 events per hour which was significantly higher than the other groups (*P* < 0.05 for trend). Pearson correlation analysis showed that only LDL-C was positively correlated with AHI events (*r* = 0.685, *P* < 0.05). Logistic regression analysis revealed that in unadjusted model, compared to dyslipidemia plus no-OSA group (reference group), OSA enhanced ASCVD risk in subjects with dyslipidemia, regardless of OSA severity. After extensively adjusted for confounding variables, the odds of dyslipidemia plus mild-OSA was reduced to insignificance. While the effects of moderate- and severe-OSA on promoting ASCVD risk in subjects with dyslipidemia remained significant, with severe-OSA most prominent (odds ratio: 1.52, 95 % confidence interval: 1.13–2.02).

**Conclusion:**

OSA combined dyslipidemia conferred additive adverse effects on cardiovascular system, with severe-OSA most prominent.

## Background

Obstructive sleep apnea (OSA) which is featured by intermittent hypoxemia during sleep is associated with atherosclerotic cardiovascular diseases (ASCVD) as revealed by previous epidemiological researches [1–3]. Owing to the increased pandemic of obesity [4, 5], a major risk factor of OSA, the incidence and prevalence of OSA is increased dramatically in recent decades [6]. Therefore, it is clinically relevant and important to screen, diagnose and treat OSA timely so as to reduce the ASCVD risk.

Dyslipidemia, as mainly defined by increased serum total cholesterol (TC) and low density lipoprotein cholesterol (LDL-C) levels, is another major risk of ASCVD. And a substantial amount of epidemiological and interventional researches have consistently demonstrated a linear relationship between serum LDL-C level and incident ASCVD [7]. In brief, LDL-C elevation is largely associated with increased consumption of saturated fat, which could also lead to central adiposity and obesity [8]. Therefore, it is conceivable that obesity may play overlapped roles between OSA and dyslipidemia, and OSA plus dyslipidemia may confer additive adverse effects on cardiovascular system. However, the evidence is lacking.

In our present cross-sectional research, we initially investigated the relationship between index of OSA and serum lipid profiles, and then evaluated the effects of OSA plus dyslipidemia on the prevalence of ASCVD. We hoped that information from our current study would broaden our understanding about the effects of OSA plus dyslipidemia on cardiovascular system, and additionally provide basics and insights for future clinical studies in managing OSA and dyslipidemia effectively so as to further reduce ASCVD risk.

## Methods

### Studied subjects enrollment

Present study was approved by the ethic committee of Shanghai Changzheng Hospital. Subjects were enrolled after informed consent was obtained, and the time of enrollment was from October of 2014 to October of 2015. All subjects had not been previously diagnosed as OSA, but were diagnosed as dyslipidemia according to self-report, or treatment with statins, or increased serum TC and/or LDL-C level.

### Clinical characteristics and laboratory data collection

Clinical characteristics including age, gender, smoking status, height and weight for calculating body mass index (BMI), waist and hip circumference for calculating waist-hip ratio, systolic/diastolic blood pressure (SBP/DBP) and heart rate (HR) at rest were recorded in electronic case report form. ASCVD included previously diagnosed as coronary heart disease (CHD), ischemic stroke and peripheral artery disease (PAD) based on clinical symptoms plus objective evidence such as coronary angiography, computer tomography scan and Doppler ultrasound. Medical history and medicine usages were also collected. Laboratory data included fasting lipid profiles and plasma glucose (FPG), glycated hemoglobin (HbA1c) and C-reactive protein (CRP) were collected and double-checked by two working staffs.

### OSA diagnosis with polysomnography

All enrolled subjects were underwent attended polysomnography and based on apnea-hypopnea index (AHI), those with AHI ≥ 5 events per hour were diagnosed as OSA [9]. In brief, AHI with 5–14.9 was defined as mild, 15–29.9 moderate and ≥ 30 severe, and less than 5 was without OSA.

### Statistical analysis

Continuous variables are presented with mean and SD and categorical variables are presented with the number and percentages. Statistical significance of differences is analyzed using one-way ANOVA or Mann–Whitney U test for continuous variables and the chi-square or Fisher exact test for categorical variables. Pearson correlation analysis was performed to evaluate the relationship between AHI and lipid profiles. Logistic regression analysis was applied to calculate odds ratio (OD) and associated 95 % confidence intervals (CI) of OSA plus dyslipidemia on ASCVD prevalence. Statistical analysis is computed using SPSS 16.0 (SPSS Inc, Chicago, IL). All of the statistical tests were two-sided and considered statistically significant if *P* < 0.05.

## Results

### Comparisons of clinical characteristics

Totally, 248 subjects with dyslipidemia were enrolled, and according to the severity of OSA, all subjects were classified into four groups as presented in Table 1. Compared to the other three groups, subjects in the severe OSA group were older, male predominant and had higher rate of smoking (*P* <0.05 for trend). Furthermore, subjects with severe OSA appeared to at correspondingly higher prevalence of OSA risk factors as reflected by higher BMI, waist-hip ratio, blood pressure, and higher rates of overweight and obesity (*P* <0.05 for trend). With respect to laboratory examination, serum levels of FPG, HbA1c, LDL-C and CRP were all significantly higher in severe OSA group (*P* <0.05 for trend). ASCVD prevalence including CHD, ischemic stroke and PAD was also considerably higher in subjects with severe OSA, and higher rates of medicines application might reflect the higher ASCVD risk (*P* <0.05 for trend). All these data revealed a linear relationship between OSA severity and ASCVD risk. AHI event in the severe OSA group was up to 35.4 ± 5.1 events per hour which was significantly higher than the other groups (*P* <0.05 for trend).

**Table 1:** Comparisons of clinical characteristics

Variables	Without OSA	Mild	Moderate	Severe
N	56	74	65	53
Age (years)	49.4 ± 11.6	52.2 ± 13.6	53.3 ± 12.2	55.3 ± 13.1*
Male, *n* (%)	30(53.6)	41(55.4)	35(53.8)	31(58.5)*
Smoking, *n* (%)	24(42.9)	37(50.0)	32(49.2)	28(52.8)*
BMI (Kg/m^2^)	24.2 ± 2.7	25.5 ± 3.5	25.8 ± 4.6	26.3 ± 3.9*
Overweight, *n* (%)	10(17.9)	30(40.5)	31(47.7)	27(50.9)*
Obesity, *n* (%)	0(0)	0(0)	3(4.6)	8(15.1)*
Waist-hip ratio	0.90 ± 0.03	0.92 ± 0.05	0.94 ± 0.04	0.96 ± 0.03*
SBP (mm Hg)	132.5 ± 10.2	131.7 ± 12.3	135.8 ± 11.6	138.2 ± 13.4*
DBP (mm Hg)	73.6 ± 8.8	73.2 ± 10.5	77.3 ± 9.1	81.2 ± 10.6*
HR (bpm)	74.2 ± 6.6	80.4 ± 9.2	88.4 ± 9.2	93.6 ± 11.2*
FPG (mmol/L)	6.0 ± 1.1	6.2 ± 1.7	6.4 ± 1.5	6.7 ± 1.6*
HbA1c (%)	6.0 ± 1.3	6.3 ± 1.3	6.6 ± 1.3	6.8 ± 1.5*
TC (mmol/L)	5.3 ± 0.5	5.3 ± 0.4	5.4 ± 0.5	5.5 ± 0.6
TG (mmol/L)	1.3 ± 0.4	1.4 ± 0.4	1.4 ± 0.3	1.5 ± 0.4
LDL-C (mmol/L)	3.1 ± 0.4	3.3 ± 0.4	3.3 ± 0.3	3.5 ± 0.5*
HDL-C (mmol/L)	1.0 ± 0.3	0.9 ± 0.3	0.8 ± 0.3	0.8 ± 0.4
CRP (mg/L)	9.2 ± 1.2	10.8 ± 2.4	12.7 ± 2.6	14.2 ± 3.3*
CHD, *n* (%)	6(10.7)	10(13.5)	11(16.9)	10(18.9)*
Ischemic stroke, *n* (%)	3(5.4)	4(5.4)	6(9.2)	6(11.3)*
PAD, *n* (%)	3(5.4)	3(4.1)	4(6.2)	5(9.4)*
Anti-platelet, *n* (%)	22(39.3)	34(45.9)	32(49.2)	28(52.8)*
ACEI/ARB, *n* (%)	26(46.4)	36(48.6)	34(52.3)	29(54.7)*
Statins, *n* (%)	29(51.8)	37(50.0)	35(53.8)	31(58.5)*
AHI (events/h)	3.2 ± 1.3	12.6 ± 2.3	21.2 ± 3.3	35.4 ± 5.1*

Denote: *P < 0.05 versus other groups; *bpm* beat per minute, *TG* triglyceride, *HDL-C* high density lipoprotein cholesterol, *ACEI* angiotensin converting enzyme inhibitor, *ARB* angiotensin receptor blocker

### Relationship between lipid profiles and AHI events

**Fig. 1:**
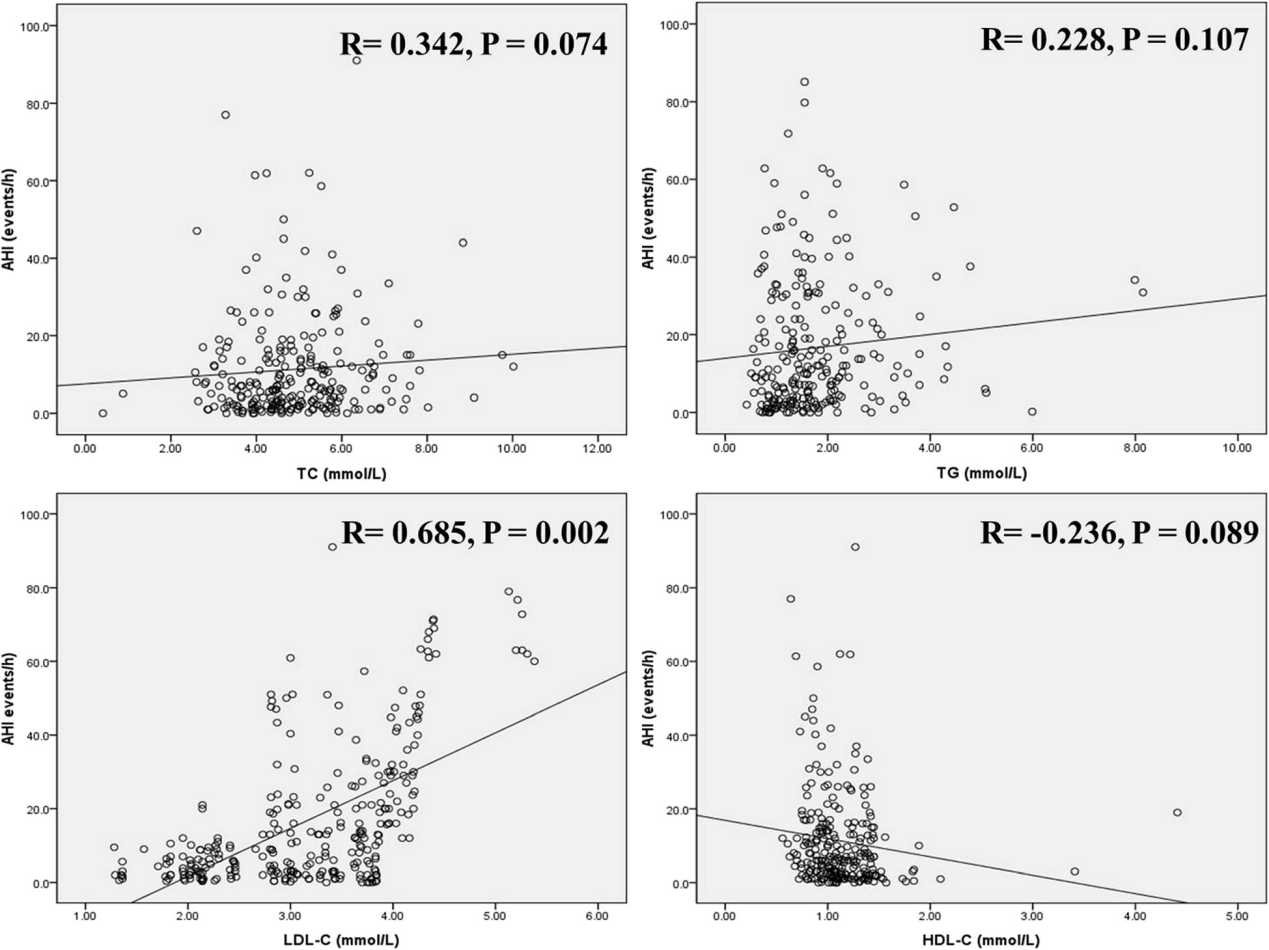
Relationship between lipid profiles and AHI events

Pearson correlation analysis was applied to evaluate the relationship between serum TC, TG, LDL-C and HDL-C levels and AHI events. As shown in Table 2 and Fig. 1 that only LDL-C were positively correlated with AHI events, with correlation coefficient was 0.685 (*P* < 0.05). And the other lipid indices were not significantly correlated with AHI events.

**Table 2:** Relationship between lipid profiles and AHI events

Variables	Correlation coefficient	*P* value
TC	0.342	0.074
TG	0.228	0.107
LDL-C	0.685	0.002
HDL-C	−0.236	0.089

### Effects of OSA plus dyslipidemia on ASCVD prevalence

Logistic regression analysis was used to analyze the effects of OSA plus dyslipidemia on ASCVD prevalence. As shown in Table 3, in the unadjusted model, compared to the dyslipidemia plus no-OSA group (reference group), OSA enhanced ASCVD risk in subjects with dyslipidemia, regardless of OSA severity. After adjusted for age, gender, BMI and waist-hip ratio (model 1), the odds of dyslipidemia plus mild-OSA on increasing ASCVD prevalence was reduced to insignificance. While the effects of moderate-OSA and severe-OSA on promoting ASCVD risk in subjects with dyslipidemia remained significant. Additional adjusted for SBP, FPG, LDL-C, CRP and statins (model 2), the effect of moderate-OSA and severe-OSA on enhancing ASCVD risk was still significant, with severe-OSA most prominent (OR: 1.52, 95 % CI: 1.13–2.02).

**Table 3:** Effects of OSA plus dyslipidemia on ASCVD prevalence

Model	Dyslipidemia + no-OSA	Dyslipidemia + mild-OSA	Dyslipidemia + moderate-OSA	Dyslipidemia + severe-OSA
Unadjusted	1	1.54(1.16–2.07)*	1.77(1.42–2.24)*	1.96(1.35–2.62)*
Mode l	1	1.27(0.89–1.84)	1.36(1.07–1.86)*	1.84(1.29–2.33)*
Mode 2	1	1.09(0.81–1.77)	1.11(0.92–1.75)*	1.52(1.13–2.02)*

Denote: *P < 0.05

Model 1 adjusted for age, gender, BMI and waist-hip ratio

Model 2 adjusted for age, gender, BMI, waist-hip ratio, SBP, FPG, LDL-C, CRP and statins

## Discussion

Dyslipidemia and OSA both are major risk factors of ASCVD, and theoretically these two co-morbidities may confer additional risk on ASCVD. Data from our current study support this concept. We observe that there is a linear relationship between OSA severity and cardiovascular risk profiles in subjects with documented dyslipidemia; secondly, there is a positive correlation between serum LDL-C level and AHI events; thirdly, logistic regression analysis shows that dyslipidemia plus OSA confer additive effect on increasing ASCVD prevalence, with severe-OSA most prominent.

With increasing pandemic of obesity, OSA has now become more prevalent and imposes great public health burden owing to its close association with ASCVD. Mechanistically, through high-frequency intermittent hypoxemia and arousals, OSA elicits systemic inflammation, oxidative stress, sympathetic nerve activation and endothelial dysfunction [10–12]. All these pathological changes are detrimental to cardiovascular system. Indeed, a substantial amount of epidemiological studies have showed that OSA is independently associated with cardiovascular events. For example, Gottlieb DJ et al. reported that OSA was a significant predictor of incident CHD and heart failure in community-dwelling middle-aged and older men [13]. In another epidemiological study, Punjabi NM and colleagues had demonstrated that OSA was associated with all-cause mortality especially that ascribed to CHD [14]. Moreover, Redline S and coworkers also had revealed that there was a strong association between AHI event and ischemic stroke in community-dwelling men with mild to moderate sleep apnea [15]. All these evidence collectively supports the notion that OSA increases risk of ASCVD.

Other than OSA, obesity is also closely associated with dyslipidemia. And the adverse effects of dyslipidemia, especially in subjects with obesity, on cardiovascular system have been consistently demonstrated in previous clinical researches [16, 17]. Nonetheless, whether OSA could enhance the detrimental effects of dyslipidemia on cardiovascular system is not fully clear yet. We therefore conducted an observational research with cross-sectional design to evaluate the effects of OSA plus dyslipidemia on ASCVD prevalence. In brief, subjects with documented dyslipidemia were enrolled and classified into four groups based on OSA degree in order to evaluate the variable effects of different OSA degree plus dyslipidemia on ASCVD prevalence. In unadjusted model, OSA, regardless of severity, rendered additional risk of ASCVD in subjects with documented dyslipidemia. Nevertheless, after extensively adjusted for potential confounding variables, only moderate-OSA and severe-OSA still had significant effects on increasing odds of ASCVD in subjects with dyslipidemia. To our best knowledge, the following aspects might partially explain these findings. On the one hand, the effects of mild-OSA which was reduced to insignificant might be due to the modest effect of OSA on cardiovascular system. Some previous epidemiological studies showed that only moderate or severe OSA had independent effects on cardiovascular system, which might support this speculation. On the other hand, it was reported that high-frequency intermittent hypoxemia contributed to dyslipidemia [18]. Therefore, severe OSA might further elevate serum LDL-C level and exaggerate the adverse effects related to dyslipidemia. Data from between-group comparison also supported this speculated as reflected by significantly higher serum LDL-C level in severe-OSA group compared to other groups. Last but not the least, since both OSA and dyslipidemia are associated with systemic inflammation as reflected by increased serum CRP level [19, 20]. And it had been demonstrated that increased serum CRP level is associated with increased risk of ASCVD [21]. Therefore, through concurrently increasing serum CRP level, dyslipidemia plus OSA might enhance ASCVD risk in an OSA-severity dependant manner.

There were some limitations of current study meriting address here. Firstly, the nature of cross-sectional design could not allow us to draw causal relationship between OSA plus dyslipidemia and ASCVD prevalence. Secondly, relative small sample size might not allow us to identify other potential differences between groups. Thirdly, despite extensively adjusted for potential confounding variables, the unrecognized biases regarding our findings still could not be ruled out.

## Conclusion

Our preliminary research showed that OSA increased ASCVD prevalence in subjects with documented dyslipidemia, and OSA plus dyslipidemia conferred additive adverse effects on cardiovascular system, with severe-OSA most prominent. In the future study, it is warranted to investigate whether improve OSA could reduce ASCVD risk associated with dyslipidemia.
